# Retinoic Acids in the Treatment of Most Lethal Solid Cancers

**DOI:** 10.3390/jcm9020360

**Published:** 2020-01-28

**Authors:** Lara Costantini, Romina Molinari, Barbara Farinon, Nicolò Merendino

**Affiliations:** Department of Ecological and Biological Sciences (DEB), Tuscia University, Largo dell’Università snc, 01100 Viterbo, Italy

**Keywords:** retinoic acid, solid cancer, all-*trans* retinoic acid, 9-*cis* retinoic acid, 13-*cis* retinoic acid, lung cancer, gastric cancer, liver cancer, breast cancer, colon cancer

## Abstract

Although the use of oral administration of pharmacological all-*trans* retinoic acid (ATRA) concentration in acute promyelocytic leukaemia (APL) patients was approved for over 20 years and used as standard therapy still to date, the same use in solid cancers is still controversial. In the present review the literature about the top five lethal solid cancers (lung, stomach, liver, breast, and colon cancer), as defined by The Global Cancer Observatory of World Health Organization, and retinoic acids (ATRA, 9-*cis* retinoic acid, and 13-*cis* retinoic acid, RA) was compared. The action of retinoic acids in inhibiting the cell proliferation was found in several cell pathways and compartments: from membrane and cytoplasmic signaling, to metabolic enzymes, to gene expression. However, in parallel in the most aggressive phenotypes several escape routes have evolved conferring retinoic acids-resistance. The comparison between different solid cancer types pointed out that for some cancer types several information are still lacking. Moreover, even though some pathways and escape routes are the same between the cancer types, sometimes they can differently respond to retinoic acid therapy, so that generalization cannot be made. Further studies on molecular pathways are needed to perform combinatorial trials that allow overcoming retinoic acids resistance.

## 1. Introduction

ATRA is the major biological active form of vitamin A, together with retinol and retinaldehyde. Retinol is present into the blood circulation bound to plasma retinol-binding protein (holo-RBP), which in turn links to another plasma protein, transthyretin, forming a protein-protein complex [1]. The uptake of retinol from the cells can either be made through plasma diffusion due to its lipophilic nature, or by an integral plasma membrane protein named stimulated by retinoic acid 6 (STRA6). STRA6 dissociates retinol from RBP and deliver it into the cell cytoplasm [2]. Once inside the cell, retinol is delivered by cellular retinol-binding protein type I (CRBP-I) to the metabolic enzymes that converted it into ATRA through two steps reaction. In the first step retinol is reversible oxidized in retinaldehyde mainly by cytosolic alcohol dehydrogenases (ADHs) or retinol dehydrogenases (RDHs). Moreover, RDHs are able to perform the conversion of retinaldehyde back to retinol, and the same reaction was also performed by the cytosolic retinoid-active aldo-keto reductases (AKRs). In the second step, retinaldehyde is irreversibly oxidized to ATRA by several cytosolic retinaldehyde dehydrogenases (RALDHs or ALDHs). Finally, ATRA cytosolic levels are controlled by the ATRA-degrading cytochrome P450 reductases (CYP26s) [2]. ATRA in the cell can be converted non-enzymatically to its stereoisomers of which the best studied are 9-*cis* RA and 13-*cis* RA [3]. 

ATRA influences cellular growth and differentiation by transcriptionally regulating gene expression by binding to nuclear retinoic acid receptors (RARs) and retinoid X receptors (RXRs). RARs and RXRs are both found in humans as three different subtypes, α, β, and γ, each of which with several isoforms, that can have different functions and tissues’ distribution and so, can activate different genes [4]. RXRs are known as the favoured heterodimerization partner for one-third of the total nuclear receptors, first of all RARs [5]. The ligand-activated transcription factors exert their action by biding to retinoic-acid responsive elements (RAREs) present on retinoid-responsive genes [6]. ATRA is selective for RARs, whereas 9-*cis* RA binds both RARs and RXRs [4]. Despite some studies reported that 13-*cis* RA can binds both RARs and RXRs [7], it is no clear if it needs before to be converted by intracellular stereoisomerization to ATRA or 9-*cis* RA [8]. Although the described pathway of RAR and RXR activated by ATRA or its isomers is the classical or genomic pathway, retinoic acids can link to other receptors. Some examples include peroxisome proliferator-activated receptor (PPAR) [9], estrogen-receptor α (ERα) [10], activator protein-1 (AP-1) [11], liver X receptors (LXRs) [12], and vitamin D receptor (VDR) [13]. The classical pathway is known to induce cell differentiation, cell arrest, and eventual apoptosis. Conversely, the non-genomic pathways regulated by these different receptors, can activate pathway with opposite functions than the classical one. The best-known example is the ATRA link to the PPARβ/δ receptors that trigger a pathway resulting in the up-regulation of the pro-survival genes [9]. It is important to note that the channelling towards one pathway or another can be due to retinoid-binding proteins (RBPs). RBPs solubilize retinoids in the intracellular compartments, and regulate their transport and metabolism. To resume the above example, in the classical pathway ATRA is delivered to RARs by cellular retinol-binding protein II (CRABPII), a RBP that channel it from the cytosol into the nucleus. In some cases, the CRABPII concentration may be less in comparison to another RBP, fatty acid binding protein 5 (FABP5). When the FABP5/CRABPII ratio is high, ATRA binds to FABP5 that delivered it to PPARβ/δ nuclear receptor activating the non-genomic pathway [14].

In 1995 the U.S. Food and Drug Administration (FDA) approved the oral administration of pharmacological ATRA concentration in APL patients following the results obtained from the study of Breitman and co-workers in 1980 [15], that discovered the potential of ATRA to induce in vitro differentiation of APL derived cells, and the subsequent human trial performed in 1987 by Huang and colleagues which proved that ATRA induces complete remission in patients with APL [16]. The treatment of APL through the oral administration of pharmacological ATRA concentration is used as standard therapy still to date [17]. Molecular studies explained that that most APL cases are characterized by some chromosome translocations that create chimeric fusion-proteins the most common of which is the PML-RARα fusion protein. In these cells PML-RARα protein is present in great excess compared to wild-type RARα and being not functional, it acts as repressor, hence only pharmacological ATRA levels (45 mg/m^2^/d for adults, in comparison to 900 and 700 µg/d of average requirement for adult men and women, respectively [18], can overcome this repression, inducing ATRA target gene expression [19]. To date, APL is the only type of cancer that can reach 95% of complete remission combining chemotherapy and the natural retinoid, ATRA [17]. Some positive effects were also recorded for cutaneous T-cell lymphomas, but with the synthetic RXR-selective retinoid bexarotene, that since 2009 was approved by the FDA for cutaneous T-cell lymphomas systemic therapy [20]. 

However, despite the great remission results for APL, the chemo-preventive and therapeutic effects of natural retinoids in solid cancers are still controversial. Nakanishi and co-workers in their study showed that ATRA treatment of hepatoma cell line HepG2 induced >2-fold changes in the expression of 402 genes including 55 linked to cell-cycle regulation, cell growth or apoptosis, after 48 h treatment and before inducing growth arrest [21]. However, clinical trials failed to found comparable results to that of APL for solid cancers. Multiple causes can generate these failures. Certainly, the short half-life of 45 min in humans [22], the poor aqueous solubility under physiological conditions, its susceptibility to light, heat and oxidants [23], can limit its clinical successful in clinical therapy, even if these were not for APL. Probably, the main obstacles to the success of this therapy should be charged in the inefficient delivery to tumor site due to oral ATRA administration, inter-patients variability in plasmatic ATRA concentration and decrease after prolonged administration [24,25], cancer relapse after a brief remission, and last but not least, tumor genetic mutations which can lead to drug resistance.

In the present review the literature about the top five lethal solid cancers and retinoic acids was compared. Preclinical studies and clinical trials were evaluated about the effects that the biologically active natural retinoic acids’ ATRA, 9-*cis* RA, and 13-*cis* RA have on the top five lethal solid cancers identified by World Health Organization (WHO). The aim was to outline among different tumor types some similarities in the molecular pathways or lack of information that can be involved in the retinoic acids’ responsiveness or resistance. For this reason, synthetic retinoic acids were not evaluated, because as structurally different molecules, they might trigger completely different pathways in comparison to natural retinoic acids.

### 1.1. Top Five Lethal Solid Cancers

**Figure 1:**
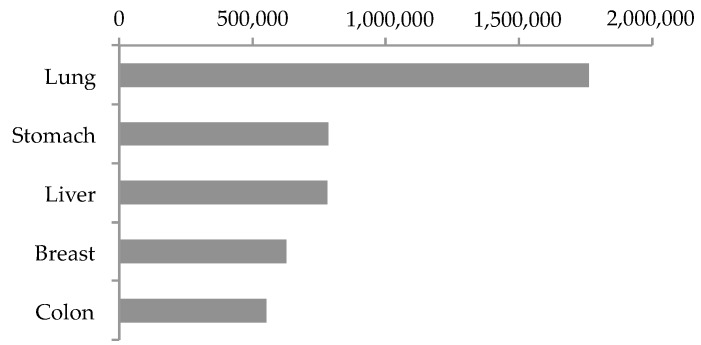
Estimated number of worldwide deaths of the top five cancers in 2018 [26].

The Global Cancer Observatory of WHO [26], estimated the top five lethal cancer sites on the basis of worldwide number of deaths in 2018 as follows from the first to fifth: lung, stomach, liver, breast, and colon (Figure 1). Noteworthy, this top five included only solid cancers, and leukaemia, with its 309,000 deaths, is located only in the eleventh place [26]. 

The first one, lung cancer, is the leading cause of cancer death worldwide, and in 2018 it caused more than 1,700,000 deaths (Figure 1) [26]. Although the high incidence of lung cancer in smokers is established, recent statistics showed an increased risk of lung cancer in those who have never smoked [27]. Lung cancer types have been subdivided into two major subtypes based on histological classification: small-cell lung cancer (SCLC) and non-small cell lung cancer (NSCLC). SCLC is the more malignant tumor, although less common. NSCLC is the most common, and is divided in three major pathologic subtypes: adenocarcinoma, squamous cell carcinoma, and large cell carcinoma [28]. The prevalence of adenocarcinoma is greatly increased in the last decades, becoming the most prevalent type of NSCLC [28]. Despite the availability of diagnostic and genetic technologies, the five-years survival rate is poor, and the reason why the mortality rates still remain higher than other cancers [29]. This is also due to the presence of cancer stem cells (CSCs) or tumor-initiating cells (T-ICs) in the tumor mass, that are a highly undifferentiated cellular population, resistant to chemotherapy and responsible to the high recurrence [30].

Similarly to lung cancer, the presence of CSCs strongly reduces the five-years survival rate also in gastric cancer [31], making it the second worldwide lethal cancer that caused more than 782,000 deaths in 2018 (Figure 1) [26]. Gastric cancer is included among the limited number of inflammation-driven cancers, highly lethal, but preventable. Indeed, among the causes the dietary factors, as nitrites and salted foods, as well as *Helicobacter pylori* infections are the most common. In recent years, improved food preservation techniques and new treatments for *H. pylori* infection allowed a gastric cancer rate decrease. However, screening programs are cost-prohibitive, so gastric cancers are routinely diagnosed only in relative advanced stages, resulting in worse outcome [32]. The WHO classified the gastric cancers in five different subtypes based on histological features: tubular, the most common; papillary, well known as liver-metastasising; and mucinous adenocarcinomas. These first three are known as well differentiated subtypes. Instead, the signet ring cell carcinoma is a malignant poorly differentiated subtype. The fifth type includes mixed carcinomas with rare variants [32]. 

Similarly to gastric cancer, the most important etiological factor that triggers liver cancers is the chronic inflammation caused by hepatitis B virus (HBV) and hepatitis C virus (HCV) infections. However, only one-third of patients are eligible for curative treatments, and even though considerable progress in diagnostic and therapeutic modalities has been accomplished in the recent years, the five-years survival rate is still 5–10% [33]. With its more than 781,000 deaths in 2018 (Figure 1) [26], liver cancer is classified as the third worldwide lethal solid cancer. Among liver cancers, hepatocellular carcinoma (HCC) is the most common (90%) malignant liver tumor [34]. Other less common subtypes included bile-duct cancer, hepatoblastoma, and various sarcomas and carcinomas. To complicate the clinical outcome, the symptoms of HCC occur mostly in the advanced stages, when HCC is highly prone to invade the portal vein causing the portal vein tumor thrombus, occurring in the 44–62% of HCC cases [35]. Such as for the other illustrated cancers, CSCs are also found in HCC [36].

**Figure 2:**
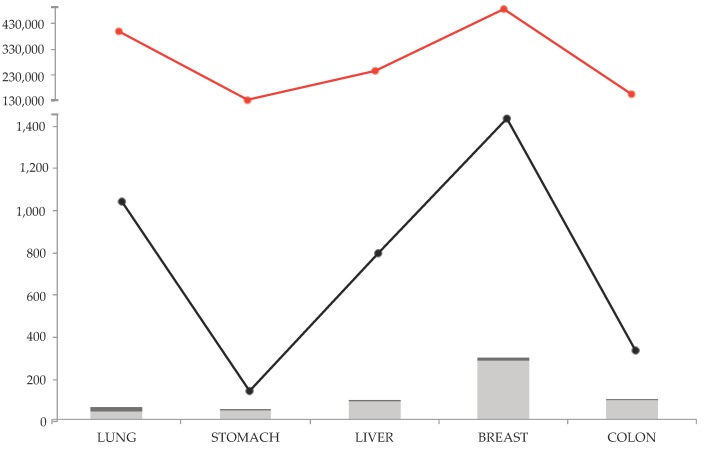
Total number of analyzed research papers. Red line: total number of papers for the corresponding cancer types. Black line: total number of papers for the corresponding cancer types and retinoic acids. Histograms: total number of research papers selected in the present review for relevancy of which: light gray, pre-clinical studies; dark gray, clinical trials.

Breast cancer remains the most common tumor found in women worldwide. It is a plethora of different malignancies that take place in the mammary gland, where carcinomas are the most common, while sarcomas and angiosarcomas have a rare incidence. The knowledge about breast cancer has enormously expanded in the last years, making it one of the best-studied lethal solid cancers and determining significant therapeutic advances (Figure 2) [37]. For this reason, and also because breast tissue is physically not a necessary organ for human survival, breast cancer patients have better survival compared to more fatal cancers. However, the great incidence and the presence of CSCs still make it the fourth lethal tumor worldwide, with more than 626,000 deaths in 2018 (Figure 1) [26]. Indeed, CSCs are resistant to currently available antineoplastic therapies, because they persistently remain in quiescent G0 phase unlike the cancer cells that quickly replicate. So, the standard therapies, which act on replicating cells, are ineffective against breast CSCs [38]. 

Finally, at the fifth place among the most fatal solid cancers there is colon cancer, with more than 550,000 worldwide deaths in 2018 (Figure 1) [26]. Often colon cancer is discussed together with rectum cancer, however splitting the data, colon cancer recorded higher mortality rate in 2018, while rectum cancer ranks the tenth place [26]. Among the colon cancer types, adenocarcinoma is the most common, followed by other less common types: colon lymphomas, gastrointestinal stromal tumor, leiomyosarcomas, carcinoid tumors, and melanomas. Recently, much has been invested to study the host-microbiota interactions, which can explain, with the concept of dysbiosis (i.e., the perturbation of the balance—eubiosis—of the different microbiota populations inhabiting the human gut), the chronic inflammation responsible of carcinogenesis and tumor progression in the colon [39]. However, as similar to what has already been discussed for the other solid cancers, despite the several advances in prevention, diagnosis, and treatment done in the last years for colon cancer, nearly 50% of patients show tumor recurrence, and in this case too, this can be attributed to the presence of CSCs [40]. 

Concluding CSCs have been found associated with the most lethal cancer types, and with features similar to stem cells, as self-renewal and pluripotent activity, they can complicate the clinical outcome inducing the major malignant phenotypes of cancers, as recurrence, metastasis and chemo-resistance. Additionally, for this reason, the action of retinoic acids, with its ability to induce differentiation, in the treatment of most lethal solid cancers needs a thorough analysis. 

### 1.2. Revision Method and Results

In order to cover all the literature in this topic, multiple databases were included in the research step. Scopus, PubMed, Web of Science were used to find over 500 research papers using the words’ combination of ‘retinoic acid’, ‘all-*trans* retinoic acid’, ’13-*cis* retinoic acid’, ‘9-*cis* retinoic acid’, and ‘lung–stomach–liver–breast–colon, cancer’. In the Figure 2 the research results are shown. The total research articles (selection for document type: article) for the considered cancer types and retinoic acids follow the trend of total research articles for each cancer types. The higher number of research articles are found for breast cancer, and the lower for stomach cancer, for both the topics ‘cancer type’ and ‘cancer type and retinoic acids’. Noteworthy, most of the found research articles are preclinical studies (light grey histograms), while very few clinical trials were found for each cancer type.

## 2. Retinoic Acids’ Action as Single Agent in Solid Cancers: Clinical Trials

As already pointed out above, there are very few clinical trials in relation to the considered solid cancers and natural retinoic acids. Among these, the clinical trials that evaluate the retinoic acids’ action as single agent against the considered solid cancers are even loss, while mostly are combinational treatment trials. 

Some early trials that analyzed the effect of ATRA in solid tumors were phase I trials established to define the maximum-tolerated dose (MTD), the dose-limiting toxicity (DLT), and pharmacokinetics of the compound in concomitant to responses at target tissues. In the first study of Lee and co-workers forty-one-recruited patients (of which 26 cases showed lung cancer) were treated with single daily oral doses of ATRA ranging from 45 mg/m^2^ to 200 mg/m^2^ for a time-lapse of 2–10 months. The found DLT was 175 mg/m^2^/day in two patients, and 200 mg/m^2^/day in two of the five that received this as maximum concentrations. For this reason the authors stated that the MTD as 150 mg/m^2^/day [41]. Similarly, in a concomitant study thirty-four mixed cancers’ patients with advanced solid tumors were treated for 29 days and the DLT was reached at 195 mg/m^2^/day [42]. A similar study was conducted also in paediatric mixed solid cancer’s patients for 28 days, and the DLT was observed at 80 mg/m^2^/day and the MTD at 60 mg/m^2^/day [43]. In the later trial of Conley and co-workers, 49 mixed cancers’ patients followed a 28 days’ therapy with a dose escalation from 45 mg/m^2^/day to 309 mg/m^2^/day. The MTD was defined as 269 mg/m^2^ per day [44]. The main toxicity observed in these studies included cheilitis skin reactions, headache, nausea, and vomiting as well as transient elevations of liver enzymes and triglyceride levels, and in the higher dosages, especially in paediatric patients, an intracranial pressure increase (*pseudotumor cerebri*). However, no objective solid tumor response, complete or partial, was observed in all the studies [41,42,43,44]. Following these studies, a phase II trial on metastatic breast cancer was done following the Lee and colleagues suggestion of 150 mg/m^2^ ATRA daily dose for 14 consecutive days. The authors found that on 14 patients that concluded the study, one patient achieved a partial response and three patients had stable disease, so ATRA did not show significant activity, but rather a high degree of interpatient variability with an acceptable toxicity [25]. 

As similar to previous phase I studies, the 9-*cis* RA actions were also evaluated in 22-mixed solid cancer’s patients with a dose escalation from 20 mg/m^2^/day to 150 mg/m^2^/day. The established DLT was 150 mg/m^2^/day causing headache and diarrhoea, and so the MTD was defined at 100 mg/m^2^/day [45]. The 9-*cis* RA toxicity and pharmacokinetics was also evaluated in paediatric patients with a dose escalation from 50 mg/m^2^/day until 110 mg/m^2^/day within 29 days. The authors found a DLT of 110 mg/m^2^/day in patients > 12 years and a DLT of 50 mg/m^2^/day in patients ≤ 12. So, the recommended MTDs were 35 mg/m^2^/day for patients ≤ 12 years, and 85 mg/m^2^/day for patients > 12 [24]. In both studies, the toxic effects are the same as found for ATRA administration, and at the same no objectives responses were observed [24,45]. 

13-*cis* RA escalating doses (from 0.5 mg/kg/day to 8 mg/kg/day or 18.5 mg/m^2^/day to 296 mg/m^2^/day, conversion made as indicated by FDA guidance 2005) [46] in 18 patients with advanced breast cancer. Toxicity and response did not change in comparison to the other retinoids [47].

All these trials pointed out that several differences between APL and solid cancers responsiveness were present, considering the resultant poor patients’ outcome, whereby, many pre-clinical studies have been accomplished to try to understand the reasons.

## 3. Pre-Clinical Studies

Early analyses consisted in in vitro treatment of cellular models to understand the responsiveness of different cancer’s types. The first pre-clinical studies in this topic, evaluated the effects that pharmacological concentrations (≥1 µM) of retinoic acids had on cancer cell lines. First investigation dates back to 1979, where different breast tumor cell lines (MDA-MB-157, 734B, SK-BR-3, and Hs578T) and one non-malignant cell line (Hs578Bst) were treated with 10 µM of ATRA. Breast cancer cell lines showed different sensitivity to this treatment. Indeed, while SK-BR-3 and 734B cell lines showed an 83% and 50% growth inhibition respectively, Hs578T was only slightly inhibited (28%), and MDA-MB-157 and Hs578Bst cell lines were not affected from the treatment [48]. Subsequent studies found similar growth inhibition results for MCF-7 and ZR-75-B cell lines. Moreover, the authors compared the treatment with ATRA and retinol, finding ATRA as a more powerful agent in inducing growth inhibition in comparison to retinol (6-700 nM for RA, against 5-8 µM for retinol) [49]. The resistance of Hs578T to 10 µM of ATRA treatment was subsequently confirmed, and similarly results were found for BT20 cell lines, both estrogen receptor negative (ER-) and not estrogen dependent [50]. 

Early analyses were also done in colon cancer cells JVC, where 1 µM of ATRA treatment induced cell morphological changes and inhibition of cell proliferation [51]. In a concomitant study Kéri and co-workers found that 10 µM of ATRA treatment decreased the proliferation rate and the tyrosine kinase activity of SW620, HT29, and COLO205, while SW480 cells showed resistance to ATRA treatment [52]. Subsequently, Reynolds and colleagues found that Moser and HT29 cell lines were susceptible to ATRA-induced differentiation, but with concentration higher than 10 µM to reach IC-50 doses. Moreover, among them Moser cell line showed less sensibility reaching an IC-50 at 70 µM [53]. 

First evidence of the 10 µM of ATRA treatment on HCC cell lines were found in the paper of Ai and colleagues, that showed the sensitivity of SMMC-7721 HCC cell line, changing their morphology from cancerous to normal [54]. Instead, the following study of Jung and colleagues analyzed the effect of ATRA treatment in some HCC cell lines. They found that ATRA dose-dependently (5–10 µM) inhibited the cell growth of HepG2 and SNU354 cell lines, but did not inhibit the growth of Hep3B and SNU449 [55]. 

Similarly, early analysis about ATRA effects on lung tumor cell lines pointed out that 17 of 22 human SCLC cell lines and 9 of 15 NSCLC cell lines exhibited resistance to growth inhibition by 1 µM of ATRA treatment. This was in contrast to normal human bronchial epithelial (NHBE) cells, which are strongly growth inhibited. Moreover, two NSCLC cell lines (H1666 and H1651), not only were resistant to growth inhibition, but actually showed growth stimulation after ATRA treatment [56]. Subsequently analysis showed that 10% fetal bovine serum supplemented medium could mask or modulate the effect of the ATRA due to the presence of various growth factors. Indeed, Wan and colleagues found that several cell lines resistant to ATRA treatment became sensitive when they were cultivated in serum-free medium. Nevertheless, Calu-1 cell line was stimulated by 10 µM of ATRA treatment also in serum-free medium [57].

Similar analyses found resistant cell lines also for gastric cancer. Early analysis on ATRA effect showed that among five different gastric cell lines, only two (SC-M1 and TSGH9201) were growth-inhibited by a concentration between 0.1 µM and 10 µM [58]. Subsequently, also the 10 µM 9-*cis* RA activity was tested on eight different gastric cell lines. All the cell lines were growth inhibited except for MKN-7 cell line [59]. In the later work of Fang and co-workers was showed that also the MKN-28 cell line was resistant to apoptosis induction after 10 µM of ATRA treatment, whereas MKN-45 was sensitive [60].

All these early studies pointed out that there are solid cancer cell line’s models that revealed retinoic acids sensitivity, but at the same time, others showed retinoic acids resistance even at higher pharmacological concentrations. Retinoic acids’ resistance is probably the reason why the chemotherapeutic potential of them was strongly limited in the previous analyzed trials. For this reason, several studies analyzed the molecular pathways and genetic modifications responsible of these different results. 

### 3.1. Defective Membrane Signaling 

The first evidence of a defective mechanism in retinol uptake was found in human colon and breast cancers, where the mRNA of STRA6 protein was found highly overexpressed [61], promoting oncogenic transformation [62]. Moreover, the hyper-activation of STRA6 was linked to Janus kinase 2/signal transducer and activator of transcription 3 (JACK2/STAT3) signaling, resulting in induction of multiple pro-oncogenic STAT target genes [62]. A subsequent study showed that down-regulation of STRA6 in colon cancer cells decreased the fraction of cancer stem cells [63]. Similarly, the recent paper of Lin and colleagues, found that STRA6 was up-regulated in gastric cancers, enhancing the proliferation and the metastasis through the Wnt/β-catenin pathway. In this in vitro model miR-873 was found down-regulated and its restoration led to its tumor suppressor action, down-regulating STRA6 [64]. However, all these papers have not investigated STRA6 oncogenic properties with retinoic acids’ treatment. 

Conversely, in the paper of Carrera and co-workers, an inverse relation between STRA6 and cell proliferation was found. Indeed, in the colon cancer cell line HCT116, STRA6 participates in p53-induced apoptosis through the elevation of intracellular reactive oxygen species, even if this was independent of the downstream activation of ATRA target genes. However, the authors highlighted that, because of ATRA-inducible gene nature of STRA6, the use of retinoids could have a positive effect on the defensive mechanism of the cell [65].

**Figure 3:**
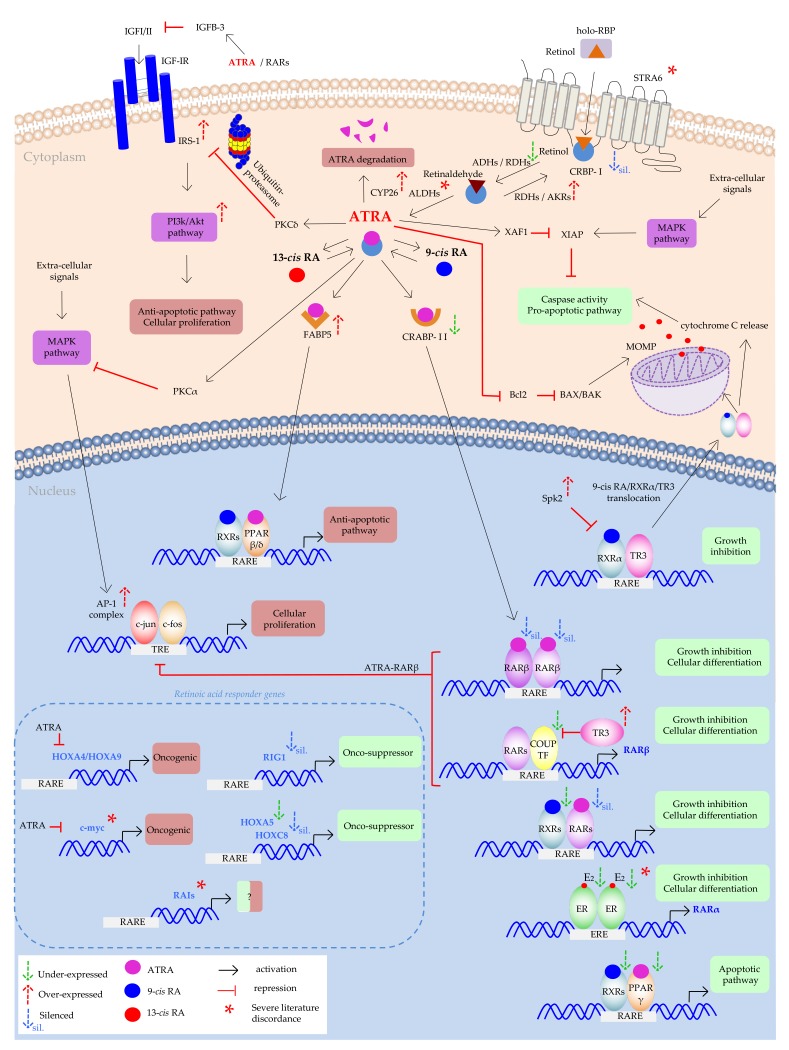
The main pathways in which retinoic acids are involved to induce cancer cell growth inhibition in ATRA-sensitive cell lines are illustrated. Moreover, molecules under-expressed, over-expressed, or silenced in ATRA-resistant cell lines are indicated. See the text for a detailed description. MOMP: mitochondrial outer membrane permeabilization.

All these evidences prove that the literature is still far from demonstrating the real action of STRA6 in solid cancers, and so further investigations are needed (Figure 3). 

### 3.2. Impaired Cytoplasmic Signaling 

#### 3.2.1. Cytoplasmic Trafficking, Defective RBPs Expression

Because of retinoic acids’ hydrophobic nature, some intracellular lipid-binding shuttling proteins must facilitate their cytoplasmic trafficking, nuclear translocation, and the delivery to nuclear receptors. However, failures in these pathways can trigger defective retinoic acids’ functions. In the first step, retinol is delivered by CRPB-I to metabolic enzymes that convert it to ATRA. Loss of CRBP-I expression was found in gastro-intestinal and breast cancers, and correlates with ATRA depletion [66,67]. Explanation of this loss of expression was provided as hypermethylation of CRPB-I gene promoter (Figure 3) [68,69]. Hypermethylation is an epigenetic mechanism that allows the transcriptional silencing of genes which is due to the alteration of DNA secondary structure and chromosome remodelling mediated by methyl-group binding proteins and histone deacetylase. In this conformation, basal transcriptional machinery has no access to DNA, and transcriptional repression occurs [70]. 

As anticipated above, when ATRA is into the cytoplasm, it mainly can follow two different pathways: the catabolic pathway through degradation by CYP proteins, or the nuclear pathway where it can perform its activity as transcription factor. As already illustrated, the main protein with this function is CRABP-II that delivers ATRA to RAR receptors. Early analysis on breast cancer cell lines showed that CRABP-II content in the cell was positively related to ATRA-growth inhibition, and that the ectopic CRABP-II enhanced ATRA action [71], and evidence showed that RARα drives CRABP-II transcription [72]. Confirmation came also by the paper of Budhu and Noy that inducing the over-expression of CRABP-II in MCF-7 cells, this dramatically enhanced their sensitivity to ATRA-growth inhibition, conversely the diminished expression rendered these cells ATRA-resistant. They explain this phenomenon with the fact that CRAPB-II delivered ATRA to RARs from cytosol to nucleus, modulating the ATRA-RARs biological activities [73]. However, as already mentioned above, CRABP-II is not the only carrier by which ATRA can enter the nucleus. In particular cases, FABP5 can perform this function, even if in normal condition ATRA has a greater affinity for CRABP-II (Figure 3). Indeed, ATRA has a KD (dissociation constant) of 0.1–0.2 nM for CRABP-II [74], in comparison to a range of 10-50 nM for FABP5. Schug and co-workers found the explanation of the ATRA-FABP5 link despite this poor affinity. They showed that in the ATRA-resistant mouse model of breast cancer MMTV-neu, the non-classical pro-survival pathway was activated by ATRA by binding to PPARβ/δ instead of RARs. Moreover, they explained this non-classical event as due to an aberrantly high intracellular lipid binding protein ratio FABP5/CRABPII [75]. So, when the FABP5/CRABPII ratio is low, ATRA binds mostly to CRABPII, which delivered it to RARs, stimulating the pro-apoptotic signal. On the contrary, when FABP5/CRABPII ratio is high, ATRA binds mostly FABP5, which targets ATRA to PPARβ/δ, activating the pro-survival genes (Figure 3) [75]. In their subsequent paper, the same authors confirmed that decreasing the FABP5/CRABPII ratio in MMTV-neu mice, ATRA was diverted from PPARβ/δ to RARs, suppressing tumor growth [75]. Following this finding, other correlations were done between FABP5 over-expression and association with poor survival and triple-negative breast cancer. Indeed, FABP5 knockdown showed a negative correlation between its level and growth inhibitory responses [76]. A subsequent study on mammary carcinoma imputed to Kruppel-like factor 2 (KLF2) the shifting of ATRA signaling from the pro-oncogenic FABP5/PPARβ/δ path to the anti-carcinogenic CRABPII/RAR pathway, inducing the expression of CRABPII and inhibiting the expression of FABP5 and PPARβ/δ [77]. It was also reported that KLF2 suppresses the expression of FABP5 in MCF-7 cell line [78]. However, a recent paper found conflicting result related to FABP5/CRABPII ratio implication on ATRA sensitivity in triple-negative breast cancer [79], so that further data are needed, especially for other solid cancer types than breast.

#### 3.2.2. Metabolic Enzymes

Considering the profound differences in ATRA response between cell lines, the metabolic pathways were analyzed to understand whether it might be due to differences in uptake and metabolism of ATRA. In a first paper the breast cancer ER positive (ER+) cell lines MCF-7 and T-47D were compared with the ER- cell lines MDA-MB-231 and MDA-MB-453. The study revealed profound differences in ATRA accumulation between ER+ and ER- cell lines, after 30 nM ATRA treatment. Moreover, ER+ cell lines showed greater amounts of ATRA metabolites in concomitant with the growth inhibitory effects, conversely to ER- cell lines that showed an accumulation of intact ATRA over longer time and no effects on growth inhibition [80]. Similarly, Hayden and Satre showed that retinol metabolism and ATRA synthesis is higher in normal human mammary epithelial cells in comparison to breast cancer cell lines MCF-7 and MDA-MB-231, suggesting that probably alterations in retinol metabolism may occur during carcinogenesis [81]. Confirmation of the previous evidence was found by Kropotova and colleagues in two different papers for gastric and colorectal cancers were an overall evaluation of gene expression in relation to the ATRA cytosolic biosynthesis were performed. In both papers the authors found a dysregulation in each step of ATRA biosynthesis with significant decreases in the mRNA levels of genes that encode the enzymes for oxidation/reduction of retinol to retinaldehyde (ADH1B, ADH1C, RDHL, AKR1B10 for both cancer types, RDH5 only in colorectal cancer, and ADH4, AKR1B, and RDH12 only in gastric cancer). Moreover, in both papers the mRNA’s decrease of RALDH1 enzyme, responsible for the irreversible oxidation of retinaldehyde to ATRA, was found. In colorectal cancer this decrease was also accompanied by an increase in the expression of the ATRA-degrading cytochrome CYP26A1, whereas in gastric cancer these enzymes were unaltered [82,83]. The same authors also correlated the decreased expression of ADH1B and ADH1C with advanced stages of colorectal carcinomas, overall indicating that dysregulation of the ATRA biosynthesis can be responsible of the cancer progression [83]. Similarly, in a later research, a different expression of genes involved in the retinoid’s metabolism was found between 44 NSCLC tumor tissues and paired normal tissues [84]. Indeed, the authors recorded a dramatic decrease in mRNA levels of genes involved in the retinol oxidation to retinal (ADH1B, ADH3, RDHL), and in the conversion of retinal to ATRA (RALDH1), with an increased expression of AKR1B10 that converts retinal to retinol, indicating that in the resistant lung tumor tissue all the pathways concur to decrease ATRA concentrations [84].

AKR up-regulation in several cancers’ types has been widely reported. First evidence showed an elevated AKR1B10 in HCC, in NSCLC cell lines, and in breast cancer’s patients [85,86,87]. AKR1C was found up-regulated in HT29 colon cancer cell line [88]. The putative mechanism is that AKR overexpressed enzymes provoke the ATRA intracellular deprivation through retinaldehyde conversion into retinol, and this condition trigger carcinogenesis events by blocking cell differentiation and promoting cell proliferation (Figure 3). In support to this hypothesis, the up-regulation of AKR enzymes was found in early stages of experimental HCC [89], and increase activity and expression of these proteins in all grading of human breast cancer’s samples in comparison to non tumor samples [90]. For this reason, some authors suggested to introduce them as early detection markers of diagnosis, staging, and prognosis [86,89]. However, even if in lung, breast, and hepatic cancers this relation is quite established, in gastric and colon cancers the topic is still open. Indeed, Ohashi and colleagues found conflicting results in comparison to previous cited of Kropotova and co-workers [82] recording a lower expression of AKR1B10 in colorectal cancers and adenomas in comparison to normal tissues [91]. Similarly, Yao and colleagues found AKR1B10 down-regulation in gastric cancer compared with paired normal mucosa, contrary to what was found by Kropotova and colleagues [83,92]. 

Conversely to what was found above in relation to the first step of ATRA metabolism, the association of impaired metabolism and solid cancers in the second step is not completely overt. Indeed, some papers support a decrease in expression of retinaldehyde dehydrogenase enzymes [82,83], until to a complete absence of ALDH6 in MCF-7 breast cancer cell line where the authors found the inability to oxidize retinaldehyde in ATRA [93]. However, other papers correlated the cancer phenotype with an over-expression of ALDH enzymes. ALDH1A1 and ALDH1A3 were found at higher levels in A459 lung cancer line [94], higher ALDH1 in gastric CSCs [95], and higher ALDH also in MCF-7/C6 CSC population selected from MCF-7 [96]. In all these papers the ATRA pharmacological treatment (10 µM) led to ALDHs’ down-regulation and CSCs’ differentiation. This discrepancy was partially explained in the paper of Coyle and colleagues, where a limit overlap between ALDH1A3-induced gene expression and ATRA-induced gene expression was found, suggesting that probably the function of ALDH1A3 in breast cancer progression extends beyond its role as retinaldehyde dehydrogenase [97]. So, research on ALDH expression and solid cancer establishment requires further investigations (Figure 3).

As reported above, the over-expression of CYP enzymes, the specifically enzymes deputed to ATRA degradation, could be the further explanation of impaired ATRA function in ATRA-resistant cell lines (Figure 3). Indeed up-regulated CYP26A1 was found in colorectal carcinoma [98], in 42% of primary breast cancers [99], and in HCC cells together with CYP26B1 up-regulation [100]. Moreover, CYP26A1 over-expression determined the tumorigenic and metastatic potential, survival characteristics reverted by its suppression [99]. Similarly, the over-expression of CYP26C1 was found in 33% of primary breast carcinomas and associated with high Ki-67 index [101].

#### 3.2.3. Escape Routes from Apoptosis

Apoptosis is a programmed cell death that results in response to a wide variety of intra- and inter-cellular stresses and insults, and it becomes necessary to stop the establishment of carcinogenesis. Retinoic acids, mainly as ATRA, has the potential to induce apoptosis in the sensitive cell lines, through the activation of either intrinsic and/or extrinsic apoptosis pathway. However, it is well known that cancer cells have evolved survival mechanisms to escape apoptotic death, and this also occurs in ATRA resistant cell lines.

ATRA can activate the extrinsic apoptosis pathway through the up-regulation of tumor necrosis factor α (TNFα), caspase-8, and death receptor Fas [102,103,104]. In sensitive breast cancer cell lines ATRA can down-regulate the expression of some anti-apoptotic molecules as Bcl-2, cdk2, cyclin D1, and survivin, that are overexpressed in one third of breast cancer types [105,106]. Other ATRA pro-apoptotic actions were found in LoVo and SW1116 colon cancer cell lines where ATRA is able to induce the expression of X-linked inhibitor of apoptosis protein (XIAP)-associated factor 1 (XAF1), a protein that functions as antagonist of XIAP by rescuing XIAP-suppressed caspase activity and inducing apoptotic growth suppression (Figure 3) [107]. Moreover, ATRA can induce the expression of some pro-apoptotic genes such as caspase 7 and caspase 9 in MCF-7 cells [108]. Cyclin D1 expression decreased in association to growth inhibition of the normal cells, and dysregulated expression of cyclin D1 resulting in inhibition of G1 phase arrest, and so, in ATRA resistance [109]. The over-expression of cyclin D1 was in part explained by mutations in β-catenin degradation pathway that led to nuclear β-catenin increased concentrations and conferred ATRA-resistance in human colon cancer cells, mechanism reverted by retinol treatment [110]. More recently another target of proliferation inhibition induced by ATRA was found in breast, liver, and gastric cancer. Indeed, the prolyl isomerase Pin1, overexpressed in many solid cancers, was found dose-dependently down-regulated after 5-10-20 µM of ATRA treatments, through the decrease of cyclin D1, β-catenin, c-myc, and CDK [111].

The activation of the onco-suppressor p53 was related to ATRA-induced apoptosis and it was found that in normal human mammary cells, apoptosis occurred after 1 µM of ATRA treatment and following to G1 phase arrest, so independent of the level of p53 expression [112]. However, conflicting results were found later in human HepG2, HCT116, and MCF-7 where a p53-dependent apoptosis was found through p14 activation. However, the same activation did not take place in Hep3B and A549 cell lines, and supposed by the authors as a lack of p53 and/or p14 [113]. Similar studies in lung cancer investigated the mechanism by which the proliferation of normal human bronchial epithelial (NHBE) cells was suppressed after ATRA treatment, and why this mechanism failed in NSCLC cells, which are derived from NHBE cells. The first study showed that 10 µM of ATRA treatment in NHBE cells suppressed their proliferation and led to G_0_ entry through the involvement of E2F transcription factor that operates as transcriptional suppressor of genes that induce cell cycle progression [114]. After that, it was showed that also the expression and activity of cyclin D1, cyclin E, cyclin-dependent kinase-2 (CDK-2), and cyclin-dependent kinase-4 (CDK-4), implicated in cell cycle progression, was inhibited by 1 µM of ATRA treatment following post-translational mechanism, which in the case of CDK-4, involved the ubiquitin-proteasome pathway. The same authors showed that the same events did not take place in ATRA resistant NSCLC cells [115]. 

Studies on A549 cell line, highly invasive, metastatic and resistant to proliferative and survival inhibitory effects of ATRA have highlighted the presence of others molecular actors implicated in ATRA resistance. In the study of García-Regalado and colleagues was found that the 5 µM of ATRA treatment in A549 cells promoted the phosphoinositide 3-kinase/protein kinase B (PI3k-PKB also named PI3k-Akt) anti-apoptotic pathway activation through the interaction between RARα-Akt. The activation of the PI3k-Akt pathway led to cell survival, invasion, and decreased expression levels of the tumors suppressors RARβ2 and p53 [116]. After that, the same group found, in the same research conditions (5 µM of ATRA treatment in A549 cells), that ATRA activates also the extracellular signal-regulated kinase (ERK) pathway in an unknown modality that, in any case, included the involvement of RARα and PI3k [117]. The inhibition of the ERK pathway, in the presence of ATRA treatment, decreased cell proliferation [117]. Similarly, in the paper of Al Wadei and co-workers, it was found that different cell lines differently respond to low dosage of 13-*cis* RA and 9-*cis* RA. Indeed, the authors found that in the BEAS-2B and NCI-H69 cell lines the treatment with 13-*cis* RA and 9-*cis* RA increase cellular cyclic adenosine monophosphate (cAMP), resulting in the activation of the protein kinase A (PKA) and the following inhibition of ERK1/2 phosphorylation resulting in the inhibition of cell proliferation. On the contrary, in the HPL1D and NCI-H322 cell lines the treatment gives the opposite effects, with the increase of ERK1/2 phosphorylation and subsequent cell proliferation [118]. 

PKC is a family of lipid-dependent serine/threonine kinases that play several cytoplasmic roles in signal transduction pathways involved in proliferation, apoptosis, and carcinogenesis. However, still nowadays the nature and the extension of the interaction between PKC proteins and retinoic acids are not well understood. First evidence showed that ATRA is able to induce PKCα expression and PKCζ repression in ATRA sensitive T-47D breast cancer cell lines resulting in anti-proliferative actions. However, in the ATRA resistant MDA-MB-231 breast cancer cell lines ATRA is not able to perform the same PKC modulations [119]. A subsequent study on the same in vitro cell line models imputed the ATRA action in inhibiting cell proliferation through PKCα and RARα activation and synergy to inhibiting the MAP kinases and c-fos induction (Figure 3) [120]. Similarly to PKCα, the activation of PKCδ was related to growth inhibition of MCF-7 ATRA-sensitive breast cancer cell line [121], and the pharmacological inhibition of both PKCα and PKCδ impaired the RARs activation by ATRA in SKBR3 human mammary cell line [122]. 

Finally, 9-*cis* RA developed an exclusive mechanism to induce apoptosis. Indeed, in gastric and breast cancer cells it was found that RXRα is able to form a heterodimer with the orphan receptor TR3 (also known as NGFI-B and nur77) in the nucleus, and when the ligand 9-*cis* RA link to RXRα, all the heterodimer 9-*cis* RA/RXRα/TR3 co-translocated in the cytoplasm and then localized in the mitochondria [123]. Here, TR3 and not RXRα, is the critical factor for inducing apoptosis [124]. Indeed, it binds Bcl-2, and inducing Bcl-2 conformational change determining its conversion from anti-apoptotic to pro-apoptotic protein triggering the cytochrome c release and apoptosis [125]. However, it was found that resistance mechanism was developed also in this pathway. Indeed, sphingosine kinase 2 (SphK2) over-expression was found in colonic cancer cells resistant to retinoic acids [126]. The SphK2 mechanism of action induced the increase of RXRα translocation into the cytoplasm and then the rapidly degradation through the polyubiquitination pathway, so inhibiting the triggering of apoptosis (Figure 3) [127].

##### Insulin-Like Growth Factors (IGFs) Pro-Survival Action

IGFs I and II, and insulin are showed to stimulate the proliferation of several breast cancer cell lines. Some works demonstrated that pharmacological ATRA concentration (greater than 10 µM) completely inhibited IGF-I-stimulated cell growth. Instead, lower ATRA concentration increased cell growth, stimulating IGF-induced proliferation [128,129,130]. Later, the explanation of ATRA inhibitory action on IGF signaling came from two papers that found an under-expression of IGF-binding protein 3 (IGFBP-3), a negative regulator of growth, in MCF-7 cell line: the ATRA treatment induces the expression of RARβ, that is directly involved in the expression of IGFBP-3 [131,132]. Similarly, Murakami and co-workers found an involvement of RARα in the IGFBP-3 up-regulation in HCC [133]. The IGFBP-3 expression after ATRA treatment was confirmed later in the paper of Dokmanovic [134]. Indeed, with the aim to understand the molecular basis that induced inhibition of cell growth in ATRA-sensitive breast cancer cell lines and why these mechanisms failed in ATRA-resistant cell lines, the induction of gene expression after 0.1 µM of ATRA treatment was investigated in MCF-7 cell line. 13 genes showed strong induction, and four of these have anti-proliferative activity: IGFBP-3, EPLIN, βIG-H3, and FAT10. Interestingly, only one gene about 13 contained a retinoid response element in its promoter, indicating that these genes are indirectly induced by retinoids [134]. Subsequently, del Rincón and colleagues tried to explain the molecular mechanism by which ATRA suppress IGFs proliferative actions. They explained that IGF-I/-II normally binds to type I IGF receptor (IGF-IR), which mostly activates the adaptor protein insulin receptor substrate-1 (IRS-1), also found over-expressed in breast cancers. The interaction of IRS-1 with p85 leads to the activation of PI3-kinase and the serine/threonine protein kinase AKT, which elicit the anti-apoptotic pathway (Figure 3). ATRA dose-dependently (0.1–100 µM) inhibited this pathway decreasing IRS-1 concentration in ATRA-sensitive breast cancer lines (MCF-7, T47-D, and ZR75-1) but not in ATRA-resistant cell lines (MDA-MB-231, and MDA-MB-468), and only over-expression of IRS-1 in presence of ATRA reactivated the ATRA-resistance mechanism [135,136]. Subsequently, the same authors explained the ATRA regulation of IRS-1 level by the ubiquitin-proteasome pathway, through PKCδ activation in the ATRA-sensitive breast cancer cell lines [137]. Conflicting results were successfully being obtained for IGFBP-3. Indeed, its elevated expression was associated with a more aggressive breast cancer phenotype and ATRA resistance. The confirmation came from the paper of Schedlich and colleagues, that immune-neutralizing IGFBP-3 in the ATRA resistant Hs578T and MDA-MB-231 cell lines restored the 10 µM ATRA-sensitivity. They explained that IGFBP-3 blocked the formation of the RAR/RXR heterodimers, and reduced the RARE-mediated transactivation of target genes [138]. However, even if for breast cancer the molecular pathway has been poorly outlined, studies are more or less missing for the other in question solid cancers. Only few papers found other IGFB proteins in gastric and colon cancer cell lines, whit different functions [139,140]. For example, in the CaCo-2 cell line was found that 1 µM of ATRA decrease cell proliferation and this was associated with a decrease of IGFB2 and IGFB4, and an increase of IGFB6 [140]. However, the mechanism and function remain to be elucidated.

### 3.3. Impaired Nuclear Receptor Signaling 

#### 3.3.1. Loss of RARβ Expression

The first observation that several lung cancer types are characterized by the loss of the short arm of chromosome 3p, where the RARβ gene is localized, led to the investigation of retinoic acid receptors functionality in relation to the different responsiveness to retinoic acids’ treatments [141]. RARβ has four isoforms with different affinities to retinoids and different biological functions [142]. Subsequent studies sometimes specifically attributed to the RARβ isoform 2 this function. Houle and co-workers proposed for the first time in 1993 that nuclear RARβ functions as tumor suppressor gene in lung tumorigenesis [143]. 

Early analysis found a down-regulation of RARβ expression in HCC cell lines [144]. Conflicting results were found later comparing HCC tissues and non-tumor tissues, where RARβ expression did not change [145,146]. Anyway, subsequent studies associated the down-regulation of RARβ expression in HCC caused by HBV and cirrhosis [147,148].

Concurrently to the work of Houle and co-workers [143], it was found a loss of RARβ expression also in several breast cancer cell lines [149]. Confirmation about the loss of RARβ expression in breast tumor tissues comes from two different papers. In the first one, the loss of heterozygosity on chromosome 3p24, including the region coding for RARβ, was reported for breast cancer specimens and normal adjacent tissues [150]. In the second paper, the in situ hybridization failed to found RARβ in breast tumor tissue and normal adjacent tissues, and found it only in normal tissues distant from the tumor sites [151]. A later study performed on the MCF10A cell lines series (i.e., four different cell lines with different tumor progression: parental, benign, pre-malignant, and malignant) showed that the loss of retinoic acids’ inducible RARβ2 expression was associated with the tumor progression [152].

The involvement of nuclear RARβ deficiency was also demonstrated as escape route of SCLC cells to the growth inhibitory activity of ATRA. The restoration of the lost expression of RARβ in the H209 SCLC cells and treatment with 1 µM of ATRA led to the growth inhibition, and this was accompanied by increased expression of the CDK inhibitor p27KipI, decreased L-myc expression, and reduced CDK-2 activity [153]. However, the same action was not present in all the lung cancer cell lines. In the paper of Li and co-workers [154] was demonstrated that the growth inhibition of ATRA-sensitive lung cancer cells Calu-6 and H460 after treatment with 10 µM of ATRA is due to apoptosis induction. However, the apoptosis induction does not occur in ATRA-resistant lung cancer cell lines SK-MES-1, H292, and H661 at the same treatment conditions. The growth inhibitory effect of ATRA through apoptosis induction was partially explained with the RARβ different expression. However, considering that RARβ was highly expressed in the lung cancer cell line H292 and despite this, they are ATRA-resistant cells, the authors speculated that probably there were others factors differently expressed among the different cell lines [154]. This same hypothesis was in part confirmed in the paper of Wan and colleagues [155]. The authors showed that despite the lung adenocarcinoma H1792 cell line express abundant mRNA levels of RARβ, it was resistant to the growth-inhibitory effects of ATRA, suggesting probably defect in retinoid signaling. Indeed, transfection with cDNA of RXRα and RARα enhanced growth inhibition by ATRA and 9-*cis*-RA, whereas cDNA of RARγ was less effective, and cDNA of RARβ was ineffective [155]. In support of the high variability between different cell lines, the study of Choi and colleagues showed that in the H460, H1299, H1703, and A549 NSCLC cell lines, though 1 µM of ATRA and 13-*cis* RA treatments did not induce growth inhibitory effects, it restored the expression of the lacking RARβ [156]. 

Later studies correlated the ATRA resistance to the impaired RARβ in gastric, and colon cancers. In their study, Shyu and co-workers, found the expression of RARβ only in two of the five gastric cell lines analyzed, TSGH9201, and TMK-1, of which only the first one was growth inhibited by 10 µM of ATRA [58]. Following studies found a decreased RARβ expression also in gastric cancer tissues [157,158]. The study of Nicke and co-workers found an up-regulation of RARβ in HT29 colon cell line, ATRA sensitive, while failed to find it in ATRA resistant LoVo cells [159]. Similarly, ATRA induction of RARβ was observed only in the ATRA sensitive colon cell lines HCT-15 and Colo201, whereas the resistant cell lines DLD-1, HT29, and WiDr expressed RARβ only when a RARβ gene vector was introduced [160]. 

Different causes were related to the lower RARβ expression in solid cancer types. The first observation came from the paper of Côté and Momparler that found that in the DLD-1 colon cancer cell line, resistant to ATRA treatment, when ATRA is combined with 5-aza-2’-deoxycytidine, a potent inhibitor of DNA methylation, a synergistic antineoplastic effect occurred [161]. The same authors reported that DNA methylation at the level of 5-methylcytosine positioned in the region of −46 to +251 from the transcription start site of RARβ2 was the reason why the expression of RARβ in DLD-1 human colon adenocarcinoma cells was silenced [162,163]. A subsequent study also showed higher methylation of RARβ2 in tumor tissues of colon cancer mucosa, than paired normal tissue samples [164]. Similarly to colon cancer, also in breast cancer the epigenetic silencing of the RARβ promoter was correlated to the hypermethylation of this DNA portion [165,166,167]. As confirmation of this, the induction of histone re-acetylation at the RARβ promoter in breast cancer cell lines and in tumor xenograft models reactivated the RARβ transcription and obtained significant growth inhibition [165]. Some studies support this hypothesis also in lung cancer. In the paper of Suh and co-workers the explanation of the loss of RARβ expression in some lung cancer cell lines was attributed to epigenetic silencing as histone H3 acetylation and hypermethylation of the RARβ promoter. However, the authors underlined that both ATRA resistance and hypoacetylation were attributable to methylated CpG islands adjacent to RARE in the RARβ promoter, in some cell lines but not in others, suggesting that multiple mechanisms contribute to this transcriptional defect in lung cancer cells [168]. However, a following study supported the importance of this event, showing that hypermethylation of the RARβ promoter occurs as an early event in lung carcinogenesis and is one of the most frequent methylation defects in the histopathologically normal bronchial epithelium of heavy smokers [169]. Promoter hypermethylation was also detected in 64% of gastric carcinoma tissues with reduced RARβ expression [170], and mainly in poorly differentiated type of gastric carcinoma [171,172]. In relation to liver cancer, despite an early research on 51 HCC tissue samples found a less frequent RARβ promoter methylation [145], in a subsequent study, the RARβ2 promoter hypermethylation was found as induced by HBV in HCC through the up-regulation of the DNA methyltransferases 1 and 3a, resulting in the RARβ2 down-regulation [147]. Recently, papers proposed that considering the high correlation between breast cancer and RARβ2 methylation, RARβ2 might be a valuable epigenetic marker for the early detection and management for breast cancer [173,174]. Moreover, its expression was found lower in breast cancer, in comparison to normal tissue and fibroadenoma, suggesting that hypermethylation may be an initial step in breast carcinogenesis [175]. In support of this, in the paper of Barnicle and co-workers, RARβ hypermethylation was found also in epithelial cells from ulcerative colitis, making aberrant DNA methylation the probable common threat between chronic inflammation and carcinogenesis (Figure 3) [176]. 

Among other causes responsible of the loss of RARβ expression, the aberrant expression of some orphan receptors are included. The orphan receptors are receptors whose ligands are unknown. Some orphan receptors have been implicated in the regulation of the retinoid response. An example is COUP-TF (chicken ovalbumin upstream promoter-transcription factor), which can enhance the transcription induced by RARs. In the paper of Wu and colleagues was showed that COUP-TF is down-regulated in H520 and H292 ATRA-resistant lung cancer cell lines, whereas the other orphan receptor TR3 was overexpressed (Figure 3). The authors demonstrated that TR3 inhibited the COUP-TF RARE binding and that the equilibrium of TR3 and COUP-TF is crucial to the inducibility of RARβ expression and ATRA growth-inhibition, because the restoration of COUP-TF expression enhances the ATRA response of ATRA-resistant cell lines [177]. The later study of Chen and colleagues demonstrated that nicotine could abrogate the growth inhibitory effect of ATRA by suppressing its ability to induce the expression of the tumor suppressor RARβ by the induction of the orphan receptor TR3 [178]. Similarly, in their study Lin and co-workers observed that in several ATRA-resistant breast and lung cancer cell lines COUP-TF is not expressed, and that the stable expression of COUP-TF is necessary to RARβ induction, in RARα-dependent manner and through the binding of the DR-8 element present in RARβ promoter [179]. Still in breast cancer, the interaction between the nuclear protein nucleolin and COUP-TFII led to the expression of RARβ2, and the nucleolin over-expression led to the expression of RARβ2, normally reduced in breast cancer [180]. Despite the importance of COUP-TF in the RARβ expression, its role in gastric, hepatic and colon cancer has not yet been investigated.

Moreover, among the other causes responsible of the loss of RARβ expression, the existence of truncated form of RARβ specifically expressed in tumor cells, RARβ-prime (RARβ’), lacking of the N-terminal domains of beta 2 and beta 4, was also related to the ATRA resistance. Indeed, the expression of RARβ’ in MCF-7 breast cancer cell line induced the ATRA resistance, while the expression of the full RARβ in both MCF-7 and MDA-MB-231 resulted in ATRA sensitivity [181].

To date it emerges that the loss of RARβ expression primarily occurs through epigenetic silencing. However, the implication of other co-repressors and co-activators necessary to RARβ promoter activation in the other solid cancer types should be analyzed and deepened. 

#### 3.3.2. Other RARs and RXRs Defects

Although most of the studies are related to RARβ, the defects in the other retinoid nuclear receptors can also impair the responsiveness to retinoic acids treatments. In the paper of Hu and co-workers, the expression of all the RARs and RXRs were evaluated in 147 gastric cancers and compared with 51 normal gastric epithelium tissues. The findings showed that RARα, RARβ, RARγ, and RXRγ were present in significantly lower levels in the tumor tissues. Moreover, the low levels of RARα expression were related to the lower overall survival in comparison to patients with higher RARα expression [158]. However, in some cell lines, the ATRA treatment up-regulates the level of RARα inducing cell growth inhibition [182]. Similarly, in an evaluation of 42 breast cancer cell lines representative of the breast cancer heterogeneity, RARα was found as the major mediator of ATRA sensitivity [183]. Indeed, the presence of RARα over-expression in breast cancer overlapped with sensitivity to retinoic acids [184]. Moreover, as RARβ, also RARα was defined as tumor suppressor silenced by extensive cytosine methylation in the promoter responsible of RARα under-expression in MCF-7 cell line, and probably in the dysregulation of ATRA signaling [185]. In a lung cancer key research, a different expression of genes involved in the retinoid metabolism was found between 44 NSCLC tumor tissues and paired normal tissues [84]. The authors found that the mRNA levels of the nuclear receptor genes RXRγ, RARα, RXRα were significantly decreased in 80%, 65%, and 57% of tumor specimens, respectively, even at early stages. So, abnormal RAR and RXR expression in malignant tumors obviously alters cell response to treatment with retinoid acids (Figure 3). 

Conversely, an over-expression of RARα was found in 32 resected samples of HCC in comparison to 14 samples of normal liver tissue, and in vitro experiments showed that when RARα is overexpressed, HCC cell lines are growth inhibited by retinoids’ concentrations that not altered the growth of primary-cultured hepatocytes [146]. Moreover, in HCC tissues and cell lines, also the over-expression of RARγ was found and correlated with growth stimulation through the activation of Akt and NFkB pathways [186]. 

Finally, few studies investigated the RARs and RXRs expression in colon cancer cells. In the study of Lee and colleagues no differences were found in RARs and RXRs expression, and only a RARβ induction occurred after ATRA treatment and in ATRA sensitive cell lines, suggesting an exclusive role of RARβ in these sensitive colon cell lines [160]. 

#### 3.3.3. RARs vs. ER

Estrogens are steroid hormones of which the primary reproductive hormone is the 17-β-Estradiol (E_2_). Estrogen receptors (ERs), after the link with estrogens, can activate the genomic pathway functioning as transcription factors or regulate gene expression by binding other transcription factors. In some types of breast cancer ERs are lacking, probably due to aberrant gene hypermethylation [187], and named ER-. ER+ breast cancer patients can benefit of endocrine therapy and have a better outcome than ER- patients [188]. Considering the high impact that estrogens exert on breast cancer, retinoic acids responsiveness was also correlated with these types of nuclear receptors. First evidence about the correlation between ER- cell lines and ATRA resistance was found in the paper of Marth and colleagues [50]. Subsequently studies correlated ER status with the expression of RARs. The first evidence came from the paper of Roman and colleagues that found a higher expression of RARα mRNA in some ER+ cell lines (T-47D, MCF-7, MDA-MB-361, BT474, and MDA-MB-134) than ER- cell lines (HBL100, MDA-MB-231, MDA-MB-330, BT20, MDA-MB-453, and Hs578T). Meanwhile, RARγ mRNA levels were similar in all cell lines, RARβ mRNA had different expression among the analyzed cell lines, and it was never detected in MDA-MB-361, BT474, and BT20. Finally, they found that 1 µM of ATRA treatment failed to alter the expression levels of RARα and RARγ in both ER+ and ER- cell lines [189]. In the study of van der Burg and co-workers, it was reported that the unresponsiveness to 10 µM of ATRA treatment in ER- cell lines (MDA-MB-231, MDA-MB-468, BT20, and Hs578T) is largely due to the absence of functional RARs in comparison to ER+ cell lines (MCF-7, T-47D, and ZR75-1). In particular, the transfection of the studied cell lines with a RARE-tk-CAT reporter construct highlighted a strong stimulation after 10 µM of RA treatment in the MCF-7, T-47D, ZR75-1 ER+ cell lines, and at most a weak induction in the MDA-MB-231, MDA-MB-468, BT20 ER- cell lines. Conversely, in the Hs578T ER- cell line the RARE-tk-CAT was strongly activated after 10 µM of ATRA treatment, suggesting that their RARs are functional [190]. So, ER- cell lines refractory to ATRA treatment is not only related to RARs functionality. Similar results were found after 9-*cis* RA treatment about the responsiveness of ER+ and ER- cell line. Moreover, MCF-7 cells treated with 10 µM of 9-*cis* RA showed no regulation for RARα and RARβ, while RARγ was up-regulated, and RXRα was down-regulated by the treatment [191]. Conversely, in the paper of Zhao and co-workers, the expression of RXRα and RXRβ was showed to be unaffected between both ER+ and ER- cell lines, while RXRγ was not expressed. However, the levels of these transcripts did not correlate with ATRA responsiveness and were not significantly altered after 10 µM of ATRA and 9-*cis* RA treatments [192]. A later study confirmed that the activation of RARs and not of RXRs is responsible for RA-induced growth inhibition in ER+ cell lines. In the same study, it was also confirmed that the induction of RARβ by 10 µM of ATRA treatment is the responsible action that lead to the growth inhibition in ER+ cell lines (MCF-7, T-47D, and ZR75-1). Indeed, when the RARβ expression vector was introduced in the ER- cell lines (MDA-MB-231, MDA-MB-468, and BT20), these cells lost ATRA resistance. Similarly, the use of RARβ-selective antagonist molecules in ER+ cell lines led to the loss of ATRA sensitivity. In light of this, the authors also investigated the role of RARα, and they found that the induction of ATRA sensitivity in ER- cell lines using RARα expression vector actually was due to the induction of endogenous RARβ expression [193]. However, subsequent papers disagreed what found by Liu and co-worker [193], supporting the idea that RARα was the crucial receptor mediating the ATRA effects in both ER+ (T47D) and ER- (SK-BR-3) cell lines, because the single use of selective antagonist for RARβ, RARγ, and RXRα did not induce the observed biological changes showed with RARα antagonist [194,195]. Another research area tried to correlate the ATRA resistance of ER- cell lines with the estrogen receptor lack. Indeed, they found that the ER transfection of the MDA-MB-231 cell line made these cells sensitive to 1 µM of ATRA-mediated growth inhibition, express higher level of RARα and exhibited a strongly CAT activation from RARE-tk-CAT constructs in comparison to parental cell line [196]. In the paper of Rubin and colleagues, they found that the 10 µM of ATRA and 9-*cis* RA treatments in MCF-7 cell line induced down-regulation of ER mRNA and protein, and down-regulation of estrogen-responsive genes PR and pS2 [191]. In a later paper of Toma and co-workers was specified that in ER- cell lines are not completely refractory to ATRA treatment, but rather the ATRA sensitivity was obtained only at high concentration (100 µM) and after prolonged time of treatment (6–8 days) [197]. Similarly, a later study on SK-BR-3 ER- cell line showed that prolonged 2 µM of ATRA treatment led to a decrease of the telomerase activity decreasing acetylation on hTERT promoter, and thus acting as antitumor agent also in ER- cell lines [198]. In later key researches the interaction between RARs and ER was found. Indeed, it has been demonstrated that RARα, RARγ and ER can occupy the same regulatory region in the chromatin, showing that probably RARα can be an essential component to maintain ER-cofactor interactions [10,199]. In a following paper, this interaction was explained as the inhibitory activity of ATRA/RARα on epidermal growth factor receptor (EGFR) by competing with the transcription factor Sp1 for binding the same promoter fragment, whereas ER increased the EGFR expression [200]. The recent paper of Miro Estruch and co-workers showed that ATRA determined an antagonism on E_2_-induced signaling, inducing anti-proliferative effects, even if without being a direct ligand of ER [201]. However, the molecular mechanisms of this interplay are still not completely understood (Figure 3). 

Although the correlations between ER and the other solid cancers were already been discussed in the literature [202,203,204,205], the relevance of ERs and RARs crosstalk in these solid cancers are completely missing, representing a promising field of research to counteract ER proliferative activity.

#### 3.3.4. PPARs Impaired Signaling as Retinoic Acids’ Resistance Mechanism

PPAR family, constituted by three different members, PPARα, PPARβ/δ (also referred as PPARβ or PPARδ), and PPARγ, are ligand-activated transcription factors with several cellular roles. The relation of PPARγ with the retinoic acids came from the necessity of its heterodimerization with RXRs receptors, which bind to 9-*cis* RA, to be activated [206]. Furthermore, it was shown that the heterodimer transcriptional activity is maximal in the presence of both PPARs and RXRs agonists [207]. In early research papers, it was shown that PPARγ activation induces growth inhibition in colon and breast cancer cell lines [208,209]. In later papers, the inhibitory effect of PPARγ activation was shown in gastric cancer cell lines and colon cancer, and it was demonstrated that its effect was enhanced by the simultaneous addition of 9-*cis* RA, causing G1 cell cycle arrest and increase of annexin V-positive cells [210,211]. A later study showed that PPARγ–RXRα activation led to the activation of intrinsic apoptosis pathway through the involvement of p53 in breast cancer cell lines (Figure 3) [212]. However, in the paper of Allred and Kilgore the differential responsiveness to their corresponding agonists rosglitizone and 9-*cis* RA by PPARγ - RXRα was found among breast, colon, and lung cancer cell lines and correlated with the differential ratio of PPARγ and RXRα present in these cancer cell lines [213]. Later papers found that in hepatoma cell lines and tissue samples, as well as in some colon cancer cell lines and colon cancer tissue samples, an accumulation of non-functional phosphorylated RXRα was present, and interfered with the correct PPARγ - RXRα signaling, triggering the progression of these cancer cells [214,215]. Therefore, impaired PPARγ and/or RXRα might explain why in some cases retinoic acids failed to counteract tumor progression in some solid cancer cell lines. 

However, although the indirect interaction between PPARγ and retinoic acids occur and its failure could be the answer to the impaired growth inhibitory action, in the last years great attention has been given to PPARβ/δ - retinoic acids pathway. In 2003, Shaw and colleagues found that while ATRA does not activate PPARα and PPARγ, it binds PPARβ/δ, modulates the conformation of the receptor, and activates the PPARβ/δ-mediated transcription [216]. In the above-cited paper of Schug and co-workers was reported that in the ATRA-resistant mouse model of breast cancer MMTV-neu, ATRA activated the non-classical pro-survival pathway binding to PPARβ/δ instead of RARs (see Section 3.2.1; Figure 3) [75]. However, still little is known, and mostly for cytoplasmic receptors FABP5 and CRABPII. Instead, if an impaired condition due to some changing in PPARβ/δ functions exists, it is not known. 

#### 3.3.5. AP-1 Over-Expression as Retinoids’ Escape Route 

The AP-1 transcription complex consists of fos and jun families’ homodimers and heterodimers that play important role in the pre-neoplastic to neoplastic progression, by activating genes with 12-O-tetradecanoylphorbol-13-acetate (TPA)-responsive element (TRE) in their promoter region [217]. Studies showed that ATRA could exert its anti-neoplastic action by the specific inhibition of AP-1 [218]. However, also in this case, modification of this mechanism leads to ATRA inefficacy. Indeed, AP-1 over-expression was correlated with ATRA resistance in breast tumor cell lines, and some ER- cell breast cancer lines (BT20, Hs578T, MDA-MB-231, MDA-MB-468) showed this over-expression [219]. Moreover, in ATRA-resistant cell lines the c-jun [220] and c-fos over-expression [221] triggers cell proliferation. The importance of the loss of RARβ expression in breast tumor was explained in the paper of Lin and colleagues, and correlated with AP-1 over-expression. Indeed, in this paper the authors stated that RARβ strongly inhibit AP-1 activity in ATRA-independent fashion, whereas inhibition of AP-1 activity by RARα and RARγ was ATRA-dependent. Therefore, RARβ could act as tumor suppressor even in the absence of ATRA, and RARβ loss expression gives AP-1 over-expression, which could abrogate the growth-inhibitory effects of ATRA through RARα and RARγ, resulting in retinoid resistance [222]. However, retinoids showed also the capacity to inhibit the AP-1 responsive genes in ATRA-sensitive MCF-7 cell line by inhibiting MKK6/p38 and MEK/ERK signaling pathway [223].

Similarly, the lung cancer Calu-1 cell line, which was stimulated by 10 µM of ATRA treatment, showed enhanced AP-1 transcriptional activity, but not with the transactivation of nuclear retinoid receptors [57]. In support of this, the following study of Lee and colleagues [224] found that 10 µM of ATRA treatment inhibited AP-1 transcriptional activity in NHBE cells but not in tumorigenic cell lines 1170I, nor in the NSCLC cell lines Calu-1, Calu-6, SK-MES-1, and ChaGo K1. In the paper of Wan and colleagues the defect in the inhibition of AP-1 by RARβ was investigated in the H1792 lung adenocarcinoma cells, resistant to the ATRA growth-inhibitory effect, despite the abundant levels of RARβ. Contrary to what was found in the above study of Lin and co-workers on breast cancer [179], the transfection with exogenous retinoid receptors RXRα and RARα restored the H1792 growth-inhibition by ATRA and 9-*cis* RA through the antagonism of AP-1 activity, whereas RARγ was less effective, and RARβ was ineffective [155]. Evidence of RXRα involvement in relation to AP-1 inhibition was also shown in Caco2 human colon cancer cells. Indeed, it was found that the repression of RXRα phosphorylation, a malfunction associated with colorectal cancer, restored the RXRα-PPARγ heterodimer formation and led to the PPARs target genes induction and AP-1 activity suppression [215]. In the paper of Wu and colleagues the ATRA-RARs-AP1 axis was investigated in gastric cancer cells. The author found that ATRA inhibited AP-1 in BGC-823 cells, which express both the nuclear receptors RARα and RARβ, whereas ATRA failed to inhibit AP-1 activity in MKN-45 cell line, deficient of RARα and RARβ. The transfection of this resistant cell line with RARα and RARβ restored the AP-1 inhibition by ATRA, inhibiting cell growth and colony formation [225]. Therefore in general, retinoid nuclear receptors, especially RARs, are implicated in AP-1 inhibition in presence of ATRA, and defective retinoid nuclear receptors signaling and/or AP-1 over-expression can trigger a pro-survival pathway (Figure 3). However, evidence is lacking for the full pathway and AP-1 implication in hepatic cancer.

### 3.4. Retinoid-Responsive Genes 

Following the studies that highlighted the retinoic acids’ activity in suppressing the growth of different cancer cell lines, some studies focused on the genes expressed after the retinoid acids’ treatment as probably tumor suppressor genes. A recent paper of Coyle and colleagues found that between 13 triple-negative breast cancer cell lines, 1400 sites were differentially methylated between ATRA resistant and sensitive cell lines [79]. Considering that the retinoic acids can activate the transcription factors that exert their action by biding to the RARE present on the promoter of the retinoid-responsive genes, impaired transcription on these genes might determine the retinoid acids’ resistance. It must be considered that also the retinoid acids’ nuclear receptor can be retinoid-responsive genes with the RARE element, and with regard to this refer to the above Section 3.1. 

#### 3.4.1. Retinoid-Inducible Gene 1 (RIG1) Hypermethylation

Following the studies that highlighted the retinoic acids’ activity in suppressing the growth of different cancer cell lines, some studies focused on the genes expressed after the retinoid acids’ treatment as tumor suppressor genes. One of the first studies allowed identifying the RIG1 in SC-M1 CL23 gastric cancer cells after 10 µM of ATRA treatment [226], also named as retinoid acid receptor responder 3 (RARRES3) or Tanzarotene-induced genes 1 (TIG1). A subsequent study identified the retinoic acid response elements in the RIG1 promoter [227]. Investigation about the expression of this gene in different stages of colon cancer differentiation reported a positive correlation among RIG1 expression and tumor differentiation, with higher levels in normal tissues in comparison to well, moderately, and poorly differentiated tumors [228]. Similarly, RIG-1 was found down regulated in human HCC tissues, and RIG-1 deficiency was found to promote HCC carcinogenesis [229]. In breast cancer tissues a negative association between RIG1 and ER expression was found. Indeed, RIG1 down-regulation was found in breast cancer ER+ cells following ER activation through E_2_ [230]. Moreover, RIG-1 loss of expression was correlated with poor clinicopathological features, due to its capacity in inhibiting the proliferation, migration, and invasion of HCC through the down-regulation of the matrix metallo-proteinases-9 (MMP9), involved in the proteolytic degradation of the extracellular matrix [231].

For these reasons another explanation about the loss of responsiveness to retinoic acids in some cancer cell lines was imputed to the involvement of this gene. The first paper on this issue found that 1 µM of ATRA treatment leads to increase RIG1 and RARβ expression in some lung cell lines. However, only relatively high level of constitutive RIG1 expression and higher level of ATRA-induced RIG1 expression may be important for growth inhibitory effects of ATRA, suggesting that RIG1 induction may be necessary but not sufficient for conferring ATRA sensitivity to the cells [232]. Similarly, in a following paper, the loss of RIG1 expression was strongly related to RIG1 promoter methylation in 28 of 53 tumor cell lines, with a methylation density >30%, and in 53% of the primary malignancies examined. However, there was no correlation between this event and the methylation of RARβ2 [233]. Aberrant methylation of RIG1 and inactive transcription were also found in gastric cancer cell lines and tissues [66,234], but also in the non-neoplastic gastric epithelia of elderly subjects and correlated with the risk to develop gastric cancer [235]. Abnormal methylation was also found in HCC tissues than in adjacent non-cancerous tissues [236], and correlated with carcinogenesis event of the liver and biliary tract [237]. A 2017 paper correlated the epigenetic silencing of RIG1 with the over-expression of the G9a histone methyl-transferase, up-regulated in HCC (Figure 3) [238].

#### 3.4.2. Retinoic Acid-Induced (RAI) Genes: Oncogenes or Tumor Suppressors?

RAI genes were first described by Imai and co-workers [239] as a retinoic-acid regulated genes from whence derived their names. Indeed, their promoter contains RAREs, even if little is known in correlation to their induction by retinoid acids [240]. The function of these genes still remains to be understood. Really little is known, and mostly in relation to their expression in different tumor sites. Different authors described RAI2 as tumor suppressor in breast cancer. Werner and colleagues showed that the depletion of RAI2 in luminal breast cancer ER+ cells is associated with a loss of epithelial differentiation that increases invasiveness [241]. In the same paper the authors showed that ER antagonists or ATRA could induce RAI2 expression. Similarly, RAI2 was found down-regulated in colorectal cancer cells and tissues compared to normal tissues [242]. The same authors connected this event to the RAI2 promoter methylation, and suggest that RAI2 may serve as a tumor suppressor inducing cell apoptosis, and inhibiting cell migration acting as tumor suppressor by inhibiting the AKT signaling pathway [242]. 

An early study demonstrated that RAI3 expression, also named G protein-coupled receptor family C group 5 member A (GPRC5A) or RA-inducible gene 1 (RAIG1), is induced by ATRA exposure in squamous carcinoma cell lines [243]. However, on the contrary to what is shown above for RAI2, papers showed an oncogenic activity related to other RAI genes. For example, evidence described an increased expression of RAI3 in 19 of 25 primary breast cancers and 6 of 11 breast cancer cell lines in comparison to normal tissues, and the suppression of cancer cell growth after transfection with RAI3 siRNA [244]. In the paper of Wu and colleagues, it was showed that the elevated expression of RAI3 in breast cancer was correlated with defective p53. Indeed, interacting p53 with the RAI3 promoter, it repressed its expression and induced apoptosis [245]. Similarly, RAI3 over-expression was found in colon cancer patients with high recurrence risk, even if the function was unknown [246]. However, conflicting results were found for RAI3 among different cancer types. Indeed, the same gene has been shown to have a tumor suppressor role in lung cancer. RAI3 knockout mice developed 76% lung adenomas and 17% lung adenocarcinomas than wild type [247]. Moreover, the RAI3 loss of expression was subsequently confirmed in human lung adenocarcinoma [248].

Instead, oncogenic activity was showed for RAI14, also named as novel retinal pigment epithelial cell gene (NORPEG), found to be aberrantly up-regulated in lung adenocarcinoma [249], and gastric cancer [250]. Recently, high expression of RAI14 in gastric cancer tissues, compared to matched normal tissues, was related to unfavourable patients’ prognosis [251]. Moreover, RAI14 knockdown inhibited proliferation, migration, and invasion, and promoted apoptosis, through the down-regulation of Akt pathway and its downstream Cyclin D1, where the downstream target gene was RAB31 [252].

RAI gene oncogenic activity was also found in HCC, where RAI16 over-expression was related to increased cell viability and colony formation in HCC cell lines and in enhanced tumor cell growth in xenograft nude mice through MAPK/ERK and TGFβ pathways [253].

In conclusion, RAI genes activity in solid cancers represents a field of considerable interest because there is still much to be investigated in relation to their oncogenic or tumor suppressor properties, especially in relation to retinoic acids’ response after treatment at pharmacological doses.

#### 3.4.3. Homeobox (HOXs) Genes Impaired Expression

The ATRA regulation of HOXs genes expression is well known in embryonic cells [254]. However, in the last years also a correlation with solid cancers was found. First evidence was found in the paper of Chen and colleagues in 2007. The same research group had shown that HOXA5 expression is lost in > 60% of breast cancer cell lines and primary tumors. Subsequently, they correlated the HOXA5 expression with the ATRA treatment only in RARβ positive breast cancer cell lines [255]. Moreover, they located the RARE in the 3’ of the HOXA5 gene, and found that in the MCF10A, ATRA resistant breast cancer cell line, the loss of both RARβ and HOXA5 expression occurred during the neoplastic transformation. Finally, they showed that the induction of HOXA5 expression was correlated with ATRA-mediated apoptosis and cellular growth inhibition [255,256]. More recently, similar down-regulated expression was found for HOXC8 gene in breast CSCs, and associated with DNA methylation in its gene promoter and expression of miR196 family members (Figure 3) [257].

On the contrary, HOXA4 and HOXA9 over-expression was found in colon CSCs as driver of carcinogenesis. The authors showed that ATRA down-regulated HOX gene expression, which correlated with the reduction of stem cells self-renewal (Figure 3) [258]. 

As emerging from the above, the knowledge about HOX genes and solid cancers is still at its infancy. HOX expression in other cancer sites should be investigated to clarify their action in relation to a possible ATRA impaired response.

#### 3.4.4. c-myc Oncogene Over-Expression

Considering the studies which demonstrated that ATRA could induce differentiation decreasing the expression of c-myc oncogene in HL60 promyelocytic leukaemia, and the fact that in some solid cancers an increased expression of c-myc occurs commonly along the tumor progression pathway, some studies have focused on the action of ATRA on c-myc expression of solid cancers, even if still now no RARE element was identified in its sequence. The first study analyzed NCI-H82 lung cancer cell line, a culture model that mimics the tumor progression transition between SCLC and NSCLC. In this model the treatment with 1 µM of ATRA resulted in decreased cellular growth, and decreased the expression of c-myc oncogene [259]. A concomitant study on breast cancer showed that in MCF-7 cells c-myc was suppressed 45% by 0.01 nM of ATRA [260]. Similarly, an early study showed that induced pre-malignant lesions of colon cancer in mice were reduced after ATRA treatment in concomitant to reduced c-myc expression [261]. Moreover, the use of c-myc antisense DNA in combination with ATRA was more effective than the agent alone in inhibiting the growth of NCI-H82 cells [262]. However, the correct mechanism through which ATRA decreases c-myc expression in some solid cancers is still unknown, even if from this first evidence a correlation between ATRA resistance and c-myc over-expression would seem not to occur.

### 3.5. Tumor Progression: CSCs and Invasion 

Recently it was demonstrated that tumor mass is composed by a heterogeneous population of cells, in which CSCs are present. Self-renewal and asymmetrical division features characterize CSCs that are responsible of tumor mass formation, progression, and recurrence, also for their ability of chemoresistance. Noteworthy, ALDH family results overexpressed in CSCs, probably for its detoxifying drug-efflux activity, and it is used as marker for CSCs identification together with CD44^+^. For example, ALDH1 over-expression correlated with poor prognosis in breast cancer patients [263]. Indeed, CSCs with ALDH^hi^CD44^+^ were found more in MDA-MB-231 ATRA-resistant cell line than in ATRA-sensitive MCF-7 cell line [264]. In this population of ALDH^hi^CD44^+^ CSCs the ATRA treatment determined a significant initial sensitization to chemotherapy and radiotherapy, even if with no long-term effect [264]. Conversely, long-term ATRA treatment was showed to inhibit gastric CSC formation in vitro, associated with decrease in CD44, ALDH1, Ki67 (proliferation index), and proliferating cell nuclear antigen (PCNA). Moreover, ATRA determined cell cycle arrest through the up-regulation of CDK inhibitors p21 and p27, and the down-regulation of cyclins and CDK genes cell cycle driving factors [95]. Similar findings were found in colon CSCs overexpressing ALDH. Indeed, the authors found that ATRA treatment inhibited colonic CSC proliferation, decreased the ALDH^hi^ population size, induced differentiation of CSC, and reduced metastasis formation [265]. Moreover, the authors imputed the CSCs establishment due to dysregulation of ATRA signaling. Indeed, as previously described (Section 3.2.2), an over-expression of CYP26A led to an intracellular higher ATRA degradation, and this in turn, led to the feedback regulation in stimulating RARs and RXRs to increase the level of ALDH1 necessary for ATRA production. All this dysregulated mechanism led to ATRA depletion and increased cell cycling [266]. However, other ATRA mechanisms of action in inducing CSCs progression inhibition were found for other cancer types. The above was already cited as the activity of ATRA in decreasing the expression of HOXA4 and HOXA9 genes (Section 3.4.3), which found as overexpressed in colon CSCs [258]. Instead, in hepatic CSCs ATRA was found to inhibit the Wnt/β-catenin and PI3K-Akt pathways, inducing cell differentiation [266]. Finally, the action of ATRA in inhibiting the PKCζ was already discussed (Section 3.2.3). A role for PKCζ was found in directing asymmetric division to generating CSC population in breast cancer. Wu and colleagues demonstrated the ability of ATRA to inhibit this activity of PKCζ at pharmacological concentrations through the activation of miR-200c. However, some aggressive CSCs demonstrated dysregulation in this pathway, so that ATRA was enable to block their progression [267]. 

Although the metastatization process involves the most aggressive phenotype of cancer cells, in some situations ATRA is able to inhibit these populations. For example, for over 10 years, ATRA was used in combination with paclitaxel to overcome the paclitaxel-resistance after long-term chemotherapy that establishes a phenotype with higher metastatic ability. Only recently it was showed that ATRA reverse the epithelial-mesenchymal transition associated with paclitaxel resistance inhibiting NFkB, and up-regulating the gap junctions [268].

The first step of metastatization consists of invasion, and to do this proteolytic degradation of extracellular matrix occurs of which matrix metalloproteinases (MMP) are the main effectors, overexpressed during tumor progression. An early analysis on breast cancer found ATRA as inhibitor of the MMP procollagenase-1 (MMP-1), up regulated in the high invasive MDA-MB-231 cell line [269]. Later studies found an inhibitory action of ATRA on another MMP, gelatinase-B (MMP-9) in MDA-MB-231 cell line through the link of ATRA to PPARγ [270], and by regulating the tissue inhibitor metalloproteinase, TIMP-1, and NFkB expression involving the ERK-PI-3K pathway [271]. Similarly, ATRA dose-dependently (10-20-30 µM) inhibited matrix metalloproteinases-2 (MMP-2) [272]. Further studies found the role of ATRA in inhibiting the MMP matrylisin (MMP-7) in colon cancer cells and in vivo animal model of colon cancer [273]. Surprisingly, also retinol is able to induce the expression of several MMPs (MMP-1, -2, -7, -9) in ATRA-resistant colon cancer cell lines, HCT116 and SW620, indicating that ATRA-resistance is not correlated with the metastatic potential inhibition [274]. 

Other ATRA actions are related to the modulation of adhesion molecules. ATRA invasion-suppressor action was showed in MCF-7/6 cell line with a dysfunctional E-cadherin/catenin complex, which normally has adhesive function. Indeed, 1 µM of ATRA treatment induced fast MCF-7/6 cell aggregation via a protein-synthesis independent mechanism and restored the dysfunctional E-cadherin/β-catenin complex [275]. Similar ATRA modulation of the E-cadherin/β-catenin complex was found in hepatoma cells, together with the enhancement of occludin/ZO-1 complex of the tight junctions system [276]. Moreover, ATRA increases cell adhesion in ER+ cell lines MCF-7 increasing tyrosine phosphorylation of two distinct molecules, focal adhesion kinase and paxillin. However, this mechanism failed to occur in ER- MDA-MB-231, and the authors linked RARα as the major factor responsible for this event [277]. However, it was showed that ATRA could also activate a pro-invasion pathway in some triple-negative breast cancer cell lines. Indeed, it activates the Src-YAP-interleukin 6 axis in MDA-MB-231 breast cancer cells increasing their invasion potential. Contrariwise, ATRA inhibits the same axis in MDA-MB-468 cells decreasing the invasion potential [278]. So, further analyses are needed to understand what is compromised in these highly proliferating phenotypes.

## 4. Conclusions

Nowadays, solid cancers cause millions of deaths every year. Although new therapies were found in pre-clinical phases, they demonstrate a lack of efficacy in the clinical trials. Retinoic acids are among them. As seen above, even though retinoic acids are able to counteract cancer cell progression and proliferation at different levels, the most aggressive phenotypes developed escape routes to bypass this action, even at retinoic acids’ pharmacological concentrations and/or after long-term treatment (Figure 3).

However, the overall frame is still missing for the most malignant solid cancers. Indeed, several pathways are still not investigated for all the cancer types, and considering the high variability present between different histological types this appears fundamental. Only by clarifying these pathways it will be possible to design combined therapies that synergize to overcame the critical steps. In the last years some combinatorial trials were performed, but the results were not the expected ones indicating that molecular mechanisms still needed to be known in detail. Finally, toxicity and other retinoic acids non-classical or proliferating actions need to be investigated in the pre-clinical phases, in different cancer types and cell lines.
